# Association between physical activity knowledge and attitude on diabetes among normal weight and overweight/obese type-2 diabetic patients: a rural community-based cross-sectional study

**DOI:** 10.4314/ahs.v22i1.35

**Published:** 2022-03

**Authors:** Ranakishor Pelluri, Srikanth Kongara, Jithendra Chimakurthy, Vanitha Rani Nagasubramanian

**Affiliations:** 1 Department of Pharmacy Practice, Vignan Pharmacy College, Vadlamudi-522213, Guntur, Andhra Pradesh, India; 2 Department of Endocrinology and Metabolism, Endo-life Speciality Hospitals, Guntur-522001, Andhra Pradesh, India; 3 School of Pharmaceutical Sciences, Vignan’ s Foundation for Science Technology and Research (VFSTR), Deemed to be University, Vadlamudi-522213, Guntur, Andhra Pradesh, India; 4 Department of Pharmacy Practice, Sri Ramachandra Institute of Higher Education & Research (SRIHER) Deemed to be University, Porur, Chennai-600116, Tamil Nadu, India

**Keywords:** Physical activity knowledge, Attitude, Body mass index, Type-2 Diabetes Mellitus

## Abstract

**Background:**

Physical activity is one of the most important regimens for the treatment of diabetes. Hence, we aimed to examine the association between physical activity knowledge (PAK), knowledge and attitude on diabetes among rural T2DM patients.

**Objectives:**

The PAK, knowledge and attitude on diabetes were targeted to evaluate in rural Indian T2DM patients.

**Methodology:**

A cross-sectional community-based survey was carried out with eighty-four patients with known T2DM in rural population of India

**Results:**

Among 84 patients, 46 were overweight/obese and 38 patients with normal weight were participated in our study. The odds of smoking were found to be a significant socio-demographic risk factor (OR: 4.42, 95% CI 0.93–20.33 and P<0.001) compared to non-smokers. The PAK categories such as A, B & D had associated with BMI. The OR, 95% CI and P. Value are (5.610, 2.18–14.38 and P<0.001; 1.72, 0.72–4.12 and P 0.030; 2.55, 1.05–6.20 and P 0.047) except in category C. Iilliterates, low annual income, poor knowledge on T2DM and negative attitude, OR (4.50; 12.87; 10.80 and 47.66) were reported disagree or don't know with PAK questionnaire.

**Conclusion:**

The results have impact on the design of new education programs will assist in preventing and managing complications related to T2DM.

## Introduction

Type 2 diabetes mellitus (T2DM) in an ever-growing major epidemic lifestyle disease, according to the international diabetes federation (IDF), diabetes atlas (2015). It is predicted that the number of adults with diabetes will increase to more than 640 million by 2040. The numbers will be increased highly in low & middle-income countries^1^. This is probably because of the staggering rise in obesity. Diabetes has manifested as a global epidemic. The change in life expectancy and lack of improvement in healthcare are in part responsible for the astounding rise in the incidence of this disease. Even in rural India, the population is undergoing lifestyle transitions due to socioeconomic growth which can also be a reason for the increasing incidence of diabetes in rural areas^2^.

Physical activity is the cornerstone of lifestyle modification aimed at preventing and managing T2DM and its related morbidities. Physical activity has been shown to improve glycemic control via increasing insulin sensitivity and glucose tolerance^3^. The evidence from randomized control trials has been demonstrated that maintenance of modest weight loss through physical activity and low carbohydrate diet reduces thencidence of T2DM up to 40–60%^4^. This is more effective than pharmacological interventions5. The risk of mortality is also reduced in individuals with T2DM^6^. Therefore, they are required to follow certain self-care practices to achieve optimal glycemic control and prevent complications. These practices include regular physical activity, appropriate dietary practices, daily foot care practice, compliance with the treatment regimen, and tackling complications such as hypoglycemic episodes^7^. Thus, the objective of this study was (i) to assess the physical activity knowledge, knowledge & attitude on diabetes disease among the rural type 2 diabetic population, (ii) to identify the valuable demographic and lifestyle factors that possibly influence the association between physical activity knowledge (PAK) among normal weight and overweight/obese patients and (iii) to examine the association between PAK and body weight, knowledge and attitude on diabetes disease and body weight of the study participants. So, this study will serve as a benchmark for future comparisons to assess the effectiveness of any educational training program for diabetic patients.

## Materials & methods

### Participants

A rural community based cross-sectional study was conducted during the January to March-2020.The patients willing to participate in the study were either gender with T2DM. The patients with less than one year of history of diabetes, pregnant women's with diabetes and the subjects unwilling to give interview were excluded from the study.

### Materials

In our study we adopted The PAK questionnaires8, consisting of 20 questions, and these were categorized into four types (Category A, B, C & D). The Category-A questionnaires consist of (1–4) knowledge pertaining to physical activity benefits, Category-B questionnaires consist of (5–9) knowledge pertaining to health benefits of physical activity in respect of DM, category-C questionnaires consist of (10–11) knowledge pertaining to details of physical activity for diabetes treatment and category-D questionnaires consist of (12–20) knowledge pertaining to physical activity conferring health benefits. The choices of response for questionnaires were “Agree/Yes”, “Disagree/No”, and “Don't know”. The participants scored one point for each correct response and zero for an incorrect or “I don't know/No” response. The respondent's score out of each determined his or her degree of understanding of the influences of PAK on diabetes-related health.

The linguistic, validated questionnaires were tested on enrolled subjects.

Physical Activity Knowledge (PAK) questionnaires: 8.

Agree /Yes = 1 Point

Disagree/No = Zero Point

Don't Know = Zero Point

Category A. Basic understanding of physical activity benefits

1. Do you believe the physical activity of a single session of 30 min per day is sufficient (T)

2. Do you believe greater health benefits can be achieved by increasing the amount (duration, frequency, or intensity) of physical activities (T)

3. Do you believe performing physical activities only on weekends is enough to achieve health benefits? (F)

4. Do you believe performing vigorous physical activities for 3 hours once a week is enough to experience health benefits? (F)

Category B. Health benefits of physical activity in respect of diabetes

5. Do you believe physical activity most days of the week can provide health benefits? (T)

6. Do you believe physical activity alone will provide health benefits? (F)

7. Do you believe patients with Type 2 diabetes should be physically active at least 5 days a week? (T)

8. Do you believe patients with Type 2 diabetes should avoid exercising in the evening? (T)

9. Do you believe regular exercise or being physically active helps to control your diabetes? (T) Category C. Details of physical activity for diabetes treatment

10. Do you believe patients with Type 2 diabetes should have resistance training that involves all major muscle groups? (T)

11. Do you believe resistance training can improve insulin resistance and increase insulin sensitivity? (T)

Category D. Physical activity conferring health benefits

Which of the following physical activities do you believe will provide health benefits?

12. Aerobics class? (T)

13. Biking? (T)

14. Dancing? (T)

15. Household cleaning? (T)

16. Jogging/running? (T)

17. Playing a musical instrument? (F)

18. Preparing meals? (F)

19. Swimming? (T)

20. Weightlifting? (T)

T - True; F - False

1. Knowledge on Diabetes: (Yes= 1 Point; No= Zero Point) Mohan D et al[9.

1. Have you ever heard of diabetes?

2. Do you know what glucose tolerance test is?

3. Do you know how to measure diabetes?

4. Do you know diabetes can cause eye disease?

5. Do you know diabetes is a genetic disease?

6. Do you know exercise can be helpful to prevent diabetes?

7. Do you know that reducing carbohydrate intake can reduce diabetes?

8. Do you know that reducing sugar intake, reduce diabetes?

9. Do you know diabetes can be controlled by avoiding smoking?

2 Attitude on diabetes (Yes- Positive, No-Negative) (Yes= 1 Point; No= Zero Point)

1. Family /friends/ healthcare professionals expect me to do exercise

2. Family /friends/ healthcare professionals expect me to follow a diabetic diet

3. Family /friends/ healthcare professionals expect me to lose weight

4. Do you know the value of exercise

5. Do you know the value of following a diabetic diet

6. Do you know the value of losing bodyweight

7. Up to you whether to do exercise

8. Up to you whether to follow a diabetic diet

9. Up to you whether to lose bodyweight

### Statistical analysis

The data entry and analysis were performed using IBM SPSS software version 2.0. The descriptive statistics such as frequencies and percentage were analyzed using Chi-square test. The levels of PAK among subjectswere assessed between PAK questionnaires and socio-demographic variables using odds ratios (OR) and 95% confidence interval (CI). Similarly, subjects were stratified based on BMI and assessed for the association.

### Ethical consideration

Approval for this study was obtained from the Institutional Ethics Committee, Endo-life specialty Hospital (IECESH), Guntur. Participation was voluntary and written informed consent was obtained from each participant.

## Results

In our study the socio-demographic details (Table 1) of a total 84 patients with either sex comprises male 75% (n=63) & females 25% (n=21). Among them males with age of <59 years were 57.7% (n=36)and42.2% (n=27) with age > 60 years. The males with body mass index (BMI) kg/m^2^ of ≤ 24.9 kg/m2 are normal weight 52.4% (n=33) and 47.6% (n=30) of BMI ≥ 25kg/m^2^ (over weight/obese) patients. The male patients with co-morbid are 58.7% (n=37), illiterates are 23.8% (n=15) and males with occupation 60.3% (n=38), annual income < 1.5 lakhs rupees are 38.1% (n=24), 31.7% (n=20) are smokers and 41.2% (n=26) were alcoholics among the male patients. The overall responders (Agree/Yes) with PAK questionnaire are 56 (66.6) and28 (33.4) are non-responders (Disagree/Don't know/No) with PAK questionnaire. The patients who have knowledge (Yes) on diabetes are 58 numbers and 26 patients do not have knowledge (No) on diabetes disease. Comprehensively, socio-demographic variables were significant (P<0.05) except gender variability. The odds of smoking were found to be a significant socio-demographic risk factor (OR: 4.42, 95% CI 0.93–20.33 and P<0.001) compared to non-smokers. The variables such as BMI, literacy levels and alcohol consumption were also associated (P < 0.05) with gender & remaining variables do not show statistical significant association (P > 0.05) with gender.

**Table 1:** Socio-demographic characteristics of male & female participants

Variables	Male		Female		OR	95% CI	P value
	n=63	%	n=21	%			
Age							
<59 years	36	57.7	12	57.1	1.00	0.37 –2.71	0.990
>60 years	27	42.2	9	42.9			Reference
BMI (Kg/m2)							
≥25 (Overweight/obese)	30	47.6	16	76.2	0.28	0.09–0.87	0.022
≤24.9 (Normal weight)	33	52.4	5	23.8			Reference
Co-morbidities							
Yes	37	58.7	16	76.2	0.45	0.14–1.36	0.151
No	26	41.3	5	23.8			Reference
Literacy levels							
Illiterates	15	23.8	13	61.9	0.19	0.06–0.55	0.001
Literates	48	76.2	8	38.1			Reference
Occupation							
With occupation	38	60.3	10	47.6	1.67	0.61–4.51	0.307
Without occupation	25	39.7	11	52.4			Reference
Annual income							
< 1.5 lakhs (Rs.)	24	38.1	13	61.9	0.38	0.13–1.04	0.056
>1.5 lakhs (Rs.)	39	61.9	8	38.1			Reference
Smoking							
Yes	20	31.7	2	9.5	4.42	0.93–20.83	<0.001*
No	43	68.3	19	90.5			Reference
Alcohol consumption							
Yes	26	41.2	0	0.0	0.016	0.00–0.28	<0.001*
No	37	58.8	21	100			Reference
Physical activity knowledge							
No/Disagree/don't know	21	33.3	7	33.3	1.00	0.35–2.85	0.414
Yes/Agree	42	66.7	14	66.7			Reference
Knowledge on DM
Poor	42	66.7	16	76.1	0.63	0.20–1.93	0.684
Good	21	33.3	5	23.9			Reference
Attitude of DM							
Negative	43	68.2	15	71.4	0.86	0.29–2.54	0.786
Positive	20	31.8	6	28.6			Reference

The P value <0.05 were considered statistically significant

Table 2 represents association between PAK &socio-demographic variables. Allvariables are having statistical association (P < 0.05) with PAK (Disagree/don't know/No) i.e., poor PAK among the variable subjects. Illiterates, low annual income, poor knowledge on diabetes and negative attitude on T2DM of odds ratios (4.50; 12.87; 10.80 and 47.66) were reported as disagree or don't know with PAK questionnaire.

**Table 2:** Association between physical activities, socio-demographic variables, health conditions, knowledge, and attitude among study participants

Variables	Disagree/ Don't know/No		Agree/Yes		OR	95% CI	P. value
	n=56	%	n=28	%			
Gender							
Male	42	(75)	21	(75)	1.00	0.35–2.85	0.000
Female	14	(25)	7	(25)			
Age							
<59 years	36	(64.3)	12	(42.8)	2.40	0.94–6.06	0.061
>60 years	20	(35.7)	16	(57.2)			
BMI (Kg/m2)							
≤ 24.9 (Normal weight)	19	(33.9)	19	(67.8)	0.74	0.09–0.63	0.003
≥ 25 (Overweight/obese)	37	(66.1)	9	(32.2)			
Co-morbidities							
Yes	41	(73.2)	12	(42.8)	3.64	1.40–9.46	0.006
No	15	(26.8)	16	(57.2)			
Literacy levels							
Illiterates	24	(42.8)	4	(14.3)	4.50	1.37–14.69	0.012
Literates	32	(57.2)	24	(85.7)			
Occupation							
With occupation	26	(46.4)	22	(78.6)	0.18	0.06–0.52	0.001
Without occupation	30	(53.6)	6	(21.4)			
Annual income							
< 1.5 lakhs (Rs.)	34	(60.1)	3	(10.7)	12.87	3.46–47.83	<0.001*
>1.5 lakhs (Rs.)	22	(39.9)	25	(89.3)			
Smoking							
Yes	8	(14.3)	14	(50)	0.16	0.05–0.47	0.0009
No	48	(85.7)	14	(50)			
Alcohol consumption							
Yes	13	(23.2)	13	(46.4)	0.34	0.13–0.92	0.029
No	43	(76.8)	15	(53.6)			
Knowledge on DM							
Poor	48	(85.7)	10	(35.7)	10.80	3.68–31.67	<0.001*
Good	8	(14.3)	18	(64.3)			
Attitude of DM							
Negative	52	(92.8)	6	(21.4)	47.66	12.23–185.68	<0.001*
Positive	4	(7.2)	22	(78.6)			

The P value <0.05 were considered statistically significant

Table 3: Shows the relationship between BMI (Overweight/Obese and Normal weight) and categories of physical activity knowledge. The respondents for PAK questionnaire categories (A, B, C and D) were stratified based on BMI. The patients with overweight/obese were more likely to have poor (Disagree/don't know/No) PAK in all categories, except Category-C. The odds ratio (OR), 95% CI and P. Value are (5.610, 2.18–14.38 and P<0.001; 1.72, 0.72–4.12 and P 0.030; 2.55, 1.05–6.20 and P 0.047). The overweight/ obese patients had statistically significant association with poor (Disagree/don't know/No) PAK except category-C questionnaires.

**Table 3:** Association between BMI & Physical activity knowledge questionnaire categories

BMI kg/m2	Response for PAK questionnaire		OR	95%CI	p. value
	Disagree/ Don't Know/No n (%)	Agree /Yes n (%)			
PAK Category-A					
Overweight/Obese (≥ 25)	32 (69.5)	14 (30.5)	5.61	2.18–14.38	<0.001*
Normal weight (≤ 24.9)	11 (28.9)	27 (71.1)			
PAK Category–B					
Overweight/Obese (≥25)	27 (58.6)	19 (41.4)	2.73	1.12–6.66	0.030
Normal weight(≤24.9)	13 (34.2)	25 (65.8)			
PAK Category–C					
Overweight/Obese (≥25)	28 (60.9)	18 (39.1)	1.72	0.72–4.12	0.272
Normal weight(≤24.9)	18 (47.4)	20 (52.6)			
PAK Category–D					
Overweight/Obese (≥ 25)	31 (67.4)	15 (32.6)	2.55	1.05–6.20	0.047
Normal weight(≤ 24.9)	17 (44.7)	21 (55.3)			

The P value <0.05 were considered statistically significant

Table 4: Describes the knowledge on T2DM disease among overweight/obese and normal-weight patients. It shoes 80.4% of patients with overweight/ obese are having poor knowledge as compared to normal weight individuals and which is statistically significant (P 0.017) among the normal weight and overweight/obese patients. Table 5: Indicates attitude of diabetes disease among normal and overweight/obese patients, the overweight/obese patients are having a negative attitude towards diabetes disease (65.2%) as compared to normal weight (44.7%) patients and indicated that, there is no statistical association between body wight and attitude on diabetes disease (P>0.05). It was indicated that,

**Table 4:** Association between BMI & Knowledge on DM

BMI kg/m^2^	Knowledge on DM		p. value
	*Poor n* *(%)*	*Good n(%)*	
Overweight/Obese (≥ 25)	37 (80.4)	9 (19.6)	
Normal weight(≤ 24.9)	21 (55.6)	17 (44.7)	***0.017***

The P value <0.05 were considered statistically significant

**Table 5:** Association between BMI & attitude on DM

BMI kg/m^2^	Attitude of DM		p. value
	*Negative n* *(%)*	*Positive n (%)*	
Overweight/Obese (≥ 25)	30 (65.2)	16 (34.8)	
Normal weight(≤ 24.9)	17 (44.7)	21 (55.3)	0.480

The P value <0.05 were considered statistically significant

## Discussion

The present study was carried out to assess the PAK, knowledge and attitude on diabetes among normal-weight & overweight/obese patients with known diabetes residing in the rural area of Guntur, Andhra Pradesh, India. Most of the study participants were in the age range of 40–65 years old, which was nearly same populations in studies done by Priyanka and Shah et al^10^,^11^. In our study there are 33.3% of illiterates as compared to 36.6% in Shah et al^11^. Therefore, to create awareness regarding the importance of education as well as patient education and lifestyle interventions are essential to maintain good health. The overall PAK was 66.6% in our study and patients agreed with our questionnaires and which was similar to Chennai Urban Rural Epidemiology Study (CURES-9)9 and which also reflect poor knowledge and attitude on the disease 69%.53% of which was observed in the study conducted by Shah et al^11^ about sociodemographic variables. The physical activity knowledge is associated with disagree/ don't know/No among socio-demographic variables, the KAP score was significantly associated with occupation and BMI, which was similar to T. Bhurosy et al.^12^. Several studies conducted in the rural community of Bangladesh showed that there is a significant association between higher body mass index (BMI) and attitude of diabetes disease^13^, like wise our study population with obesity/overweight are had poor knowledge on T2DM, and the results were similar with the study conducted in Pretoria^14^ and they had positive attitude towards T2DM. Gul N et al^15^ were also noticed that, the knowledge, attitude and practice scores were low in their study. Those are similar with our study.

Our study has limitations due to moderate sample, which may limit generalisation of the findings. Nevertheless, there is a need of education strategies to improve PAK and knowledge and attitude on T2DM among the rural population. The efforts can improve diabetes related consequences and hazards.

## Summary

In a nutshell, the results may give a true reflection on overweight/obese patient's socio-demographic status and PAK.As evidenced by the study, it was noticed that the socio-demographic variables are statistically influenced by PAK and gender. The PAK was associated with BMI. Normal weight patients were having good PAKand also having good knowledge on diabetic disease along with they have a negative attitude on the diabetic disease. In addition to our summary, there is a much need for patient education on PAK, as well as knowledge on the diabetic disease, is very much essential in order to maintain good health. Moreover, in rural areas where education and information are not readily accessible, there is a greater chance poorer perception and practices. Therefore, efforts should be directed towards developing and making education programs to empower the personswith diabetes. This will enable the patients to change their attitude towards management of diabetes. It is hoped that these findings have major implications for the design of a patient education program which helps to prevent further complications related to the diabetic disease.
